# Osteotomy after medial malleolus fracture fixed with magnesium screws ZX00 - A case report

**DOI:** 10.1016/j.tcr.2022.100706

**Published:** 2022-10-04

**Authors:** Patrick Holweg, Viktor Labmayr, Uwe Schwarze, Nicole G. Sommer, Martin Ornig, Andreas Leithner

**Affiliations:** a Department of Orthopaedics and Trauma, Medical University of Graz, Graz, Austria; b Department of Dentistry and Oral Health, Division of Oral Surgery and Orthodontics, Medical University of Graz, Austria

**Keywords:** Magnesium implant, Medial malleolus fracture, OD Tali

## Abstract

Magnesium alloys have recently become the focus of research, as these implants exhibit suitable biocompatibility and appropriate mechanical properties (Grün et al., 2018 [1]).

Through intensive preclinical and clinical investigation, many questions regarding stability, biocompatibility and degradation behavior have been answered (Holweg et al., 2020 [2]). This case report aims to describe handling of these implants in a revision situation, especially when located in situ. To describe available options and relevant considerations, including planning and implementation, a revision surgery of a healed medial malleolus fracture is presented. A medial malleolus fracture was primarily treated by a trauma surgeon with two magnesium screws. Due to an osteochondral lesion of the talus, a revision surgery with osteotomy of the medial malleolus was necessary after 17 months. In this revision, conventional screw removal was not possible due to the degradation of the implant. Taking the degradation and the yield strength of the implant into account, we have chosen on the one hand to over-drill and on the other to leave and perforate the screw.

To the best of our knowledge, this is the first case study focusing on the clinical intraoperative site of human bone stabilized with magnesium screws. Despite the hydrogen gas production that occurs during degradation, a solid bone-to-implant interface was evident. With this report, we want to encourage the surgical user to get more involved with resorbable magnesium implants.

## Introduction

In the last decade, resorbable implant materials have become increasingly important in orthopedic surgery and traumatology [3], [4].

In addition to the advantage of resorption and the elimination of the need for metal removal, these materials must exhibit good biocompatibility, sufficient yield strength and homogenous material degradation. Among these resorbable metals, magnesium (Mg) alloys are progressively coming into the focus of research, as they possess suitable biocompatibility and appropriate mechanical properties [1], [2], [5]. The major concern about the rapid degradation has been addressed in recent years with various additives like zinc, calcium or rare earth elements to avoid uncontrolled gas evolution with subsequent gas cavity formation [6].

By now, many questions about the long-term performance of Mg implants have been answered in preclinical studies. The promising clinical results with Mg implants were displayed in several clinical studies. Herber et al. recorded adequate fracture stabilization with sufficient yield strength of the Mg-based implant ZX00, after operative treatment of medial malleolus fractures [7].

With this case report, we aim to address another aspect of these implants, describing the easy handling in a revision situation without implant removal, which in this case was necessary due to an underlying osteochondral lesion of the talus.

## Case report

**Fig. 1:**
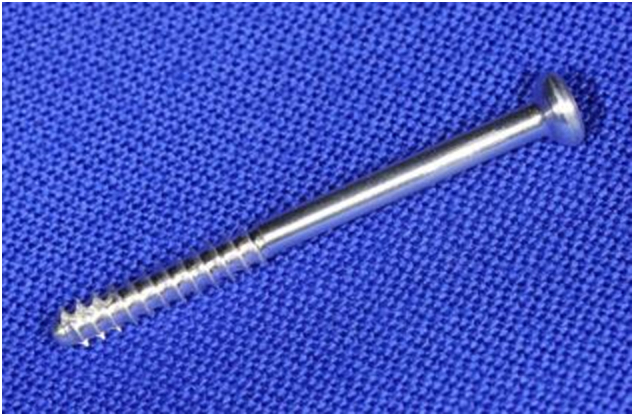
External appearance of ZX00 screw with a length of 40 mm and a diameter of 3.5 mm, partly threaded at the distal part.

This case report originated from a first in man study conducted at the Department of Orthopaedics and Trauma (approved by the ethics committee 28-071 ex 15/16) [5], including 20 patients treated with ZX00 implants for medial malleolus fracture stabilization. These ZX00 screws composed of ultra-high pure Mg (99.1 wt%) alloyed with Zn (0.45 wt%) and Ca (0.45 wt%) had a length of 40 mm and a diameter of 3.5 mm. Implants were threaded at the distal part for the use as compression screws (Fig. 1). The patient was originally treated in participation of this study.

In December 2018, the patient in question approached the authors' department for the first time and presented with pain after twisting her ankle the day before. An isolated fracture of the medial malleolus was diagnosed by X-ray.

**Fig. 2:**
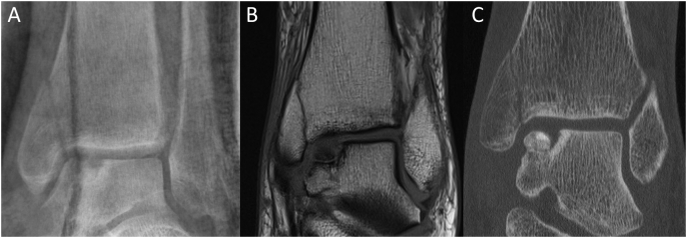
A: Anteroposterior (AP) ankle radiograph after trauma: medial malleolar fracture without displacement; bone defect at the joint line of the medial talus shoulder; B: MRI of the ankle; recent fracture of the medial malleolus and a 4th grade OD at the medial shoulder of the talus with a defect size of 9.4 × 5.7 mm; C: associated CT scan.

Furthermore, an osteochondral lesion of the medial talus shoulder was suspected on the X-ray. For clarification, a CT and an MRI of the ankle were performed, revealing a fourth degree Osteochondral lesion of the talus, beside the fracture of the medial malleolus (Fig. 2).

An osteochondral lesion, a circumscribed bone lesion below the articular cartilage, usually occurs after repetitive trauma. In this case, the osteochondral lesion must be regarded as pre-existing and independent of the actual injury to the medial malleolus. Conversely, the loss of part of the articular surface through the osteochondral lesion must be considered a contributing factor for a fracture incident in this area, after sprained ankle trauma.

With this diagnosis, the indication for surgery was made and the patient was informed accordingly. We aimed to fill the defect in the talus with an autologous iliac crest bone graft.

The zone of osteochondral defect at the talus should be approached through the existing medial ankle fracture.

### Handling of the magnesium screw ZX00 - intervention 1

The operation was performed 10 days after the initial diagnosis of the medial malleolus fracture. The medial malleolus fracture was opened with a prong, and the osteochondral lesion of the talus was exposed to the full extent. After the defect in the talus was filled with an autologous bone graft of the iliac crest, the medial malleolus fracture was anatomically reduced.

Subsequently, two parallel Kirschner wires were positioned perpendicularly to the medial malleolar fracture line for temporary fixation and as a guide for the cannulated drill. After checking the position of the wires fluoroscopically, the wires were overdrilled with a 2.7 mm cannulated drill to prepare the hole for the screws.

Drilling was performed up to the fracture line. Subsequently, the first wire was removed, and the self-tapping ZX00 compression screw was inserted into the hole.

Final fixation of the fracture was achieved with the second ZX00 screw after removal of the second wire. The insertion torque of the screws was limited at a force of 1.5 Newton-meter with a torque handle.

**Fig. 3:**
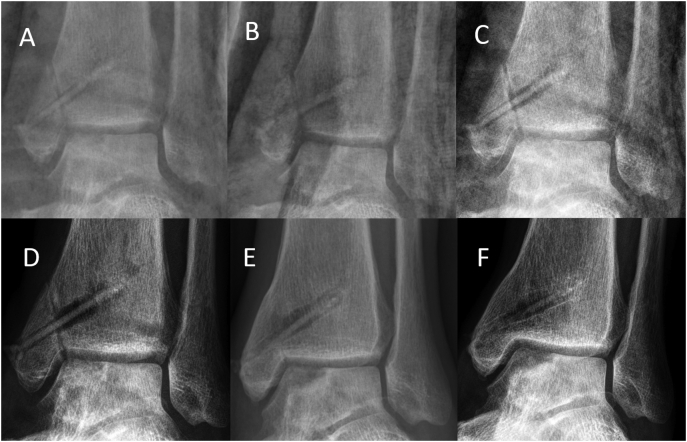
Anteroposterior (AP) radiograph of the ankle. (A) 2 weeks timepoint radiograph, anatomic reduction and stabilization of the medial malleolar fracture with two Magnesium screws; restored joint line of the talus after defect filling with autologous spongiosa. (B) 4 weeks timepoint radiograph (C) 8 weeks timepoint radiograph; visible fracture line at the medial malleolus, small signs of radiolucent zones within the bone surrounding the screws (D) 12 weeks timepoint radiograph, increasing radiolucent area around the screw (E) 24 weeks timepoint radiograph with complete fracture consolidation, decrease of radiolucency within the bone surrounding the screws (F) 52 weeks timepoint radiograph, new bone formation at the radiolucency around the screw, significant degradation of the implant.

Postoperatively, cast immobilization was performed for 8 weeks. A gradual increase in load bearing was carried out from the 9th postoperative week. In the follow-up checks at 8, 12 and 24 weeks after surgery, the patient was symptom-free with an equal range of motion on both sides of the upper ankle joint (Fig. 3).

**Fig. 4:**
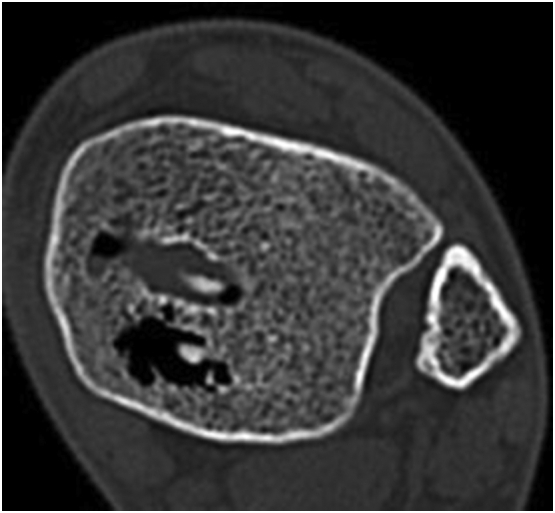
52 weeks timepoint CT scan of the ankle; new bone formation at the radiolucency around the screw, significant degradation of the implant.

During the annual follow-up, the patient reported increasing pain in the area of the medial upper ankle joint. A CT scan was performed for further diagnostic work. We discovered an increase in the size of the defect in the area of the osteochondral lesion of the talus. Furthermore, a consolidated fracture of the inner malleolus was presented (Fig. 4).

The lack of healing of the autologous graft transplant at the talus and the clinical situation of increasing pain lead to the decision of the indication for reoperation, to avoid osteoarthritic changes. The recurrence of the osteochondral lesion was independent of the consolidated fracture of the medial malleolus treated with ZX00 screws.

### Handling of the magnesium screw ZX00 - intervention 2

The second operation was performed 17 months after the first treatment of the osteochondral lesion of the talus and the medial malleolus fracture. A medial osteotomy was again planned to address the defect in the talus with an osteochondral autograft transfer system (OATS). Preceding removal of the ZX00 screws was not arranged due to the advanced resorption of the implants.

A V-shaped osteotomy was made at the level of the consolidated medial malleolus fracture, using an oscillating saw and image intensifier control. The bone and the embedded ZX00 screws were easily severed with an oscillating blade. After that, the osteotomized medial malleolus could be picked away with a prong. An inspection of the osteotomized cancellous bone revealed the ends of two ZX00 screws cut in half.

**Fig. 5:**
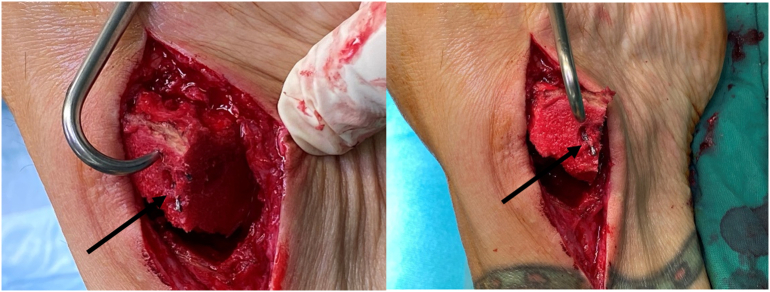
Intraoperative situs of the ankle region; osteotomized medial malleolus picked away with a prong; excellent bone to implant interface with direct contact between the cancellous bone and the screw (black arrow).

Further macroscopic examination showed a considerable reduction in the cross-section of the ZX00 implant. Despite resorption and hydrogen gas evolution, we found direct contact of the cancellous bone with the screw in most places. Consequently, the strong bone-to-implant interface impeded the screw extraction, despite resorption of the thread (Fig. 5).

**Fig. 6:**
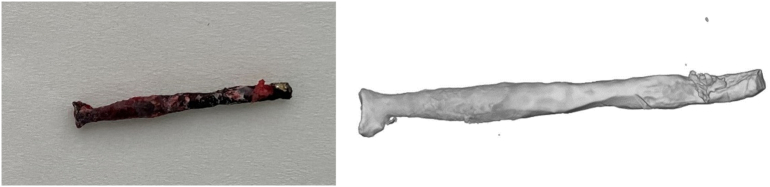
Proximal half of one magnesium screw; implant was overdrilled with a cannulated drill over its entire length in the medial malleolus and recovered in one piece. Significant reduction in the cross-section of the magnesium implant to about 50 % of the original size.

We decided to remove the two medial parts of the ZX00 screws in the distal medial ankle fragment. The two screws were overdrilled with a cannulated drill over their entire length at the medial malleolus and were recovered in one piece (Fig. 6).

**Fig. 7:**
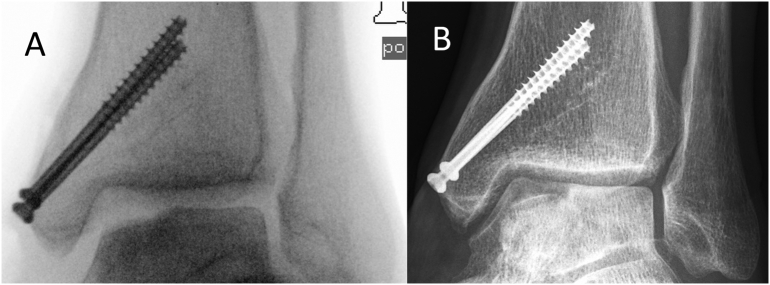
A: Anteroposterior (AP) intraoperative fluoroscopy of the ankle, anatomic reduction and stabilization of the medial malleolar fracture with two titan screws; restored joint line of the talus after defect filling without OATS plastic. B Anteroposterior (AP) x ray of the ankle one year after revision surgery; healed osteotomy of the medial malleolus with enclosed titan screws;

The screws parts in the central tibia were left in place. After filling the defect in the talus, the osteotomy was reduced and conventionally refixed with 2 cannulated titanium screws in the area of the removed and enclosed ZX00 screws (Fig. 7). This time, conventional implants had to be used as the ethics committee's approval was for primary application only.

Postoperatively, cast immobilization was performed for 8 weeks. A gradual increase in load bearing was carried out from the 9th postoperative week. In the follow-up checks at 12, 24 and 52 weeks after surgery, the patient presented an excellent clinical outcome without any pain or unrestricted movement (Fig. 7).

## Discussion

The intensive scientific work in this area has already answered many questions about the performance of Mg alloys in the clinical setting [8], [9]. Initial problems of excessively rapid degradation of pure Mg with large gas cavities in the bones were solved by adding alloying substances.

We have already described the excellent clinical applicability of the ZX00 alloy in the short to mid-term follow-up [8]. The evaluation of functional and radiological results of medial malleus fracture fixation through a prospective clinical investigation revealed promising functional results and an adequate fixation of medial malleolus fracture. All fractures were healed without secondary displacement or implant breakage. Serial radiographs exhibited a homogenous degradation of the implant [5].

The aim of this case report was to demonstrate the handling of ZX00 screws in a revision situation. In addition, the clinical intraoperative site of human bone with enclosed Mg screws became visible.

In the first intervention, the handling for inserting the ZX00 screw was analogous to the handling of conventional titanium or steel implants. After drilling of the first cortex, the self-tapping ZX00 screws were inserted without difficulties. Care was taken when tightening the screws to the bone. To avoid breakage of the screw, which has a yield strength similar to that of bone, a torque handle limited to 1.5 Newton-meter was used.

The possibilities for the surgeon in the event of an intraoperative or postoperative complication caused by an implant failure can be assessed based on the revision intervention presented in this case report. The reason for the revision was a progression of the osteochondral lesion despite autologous bone graft transplantation. The fracture stabilization with ZX00 screws revealed no complications.

If the Mg implant fails to stabilize the fracture, due to implant breakage or malpositioning, there may be problems removing the implant with the screwdriver, especially at advanced stages of implant resorption. Although the screw cannot be removed in the conventional way with a screwdriver, there are many options in revision surgery, dependent on the degree of resorption state of the implant. As shown above, there are essentially two options in this case: implant removal by overdrilling or leaving the implant in place, since the implant residuals will disappear over time. As such, they can remain in place, be drilled into, or even be replaced with another implant.

The macroscopic examination of the intraoperative sites revealed a significant reduction in the cross-section of the ZX00 implant after 17 months. Despite resorption and hydrogen gas release, there was direct tight contact of the cancellous bone with the screw in most places. This fact strengthens the hypothesis of the osteoinductive effect of Mg implants. Despite the emitted hydrogen gas, the resulting cavity filled up with new bone over time.

Importantly, this case shows that switching to conventional implant materials like Ti works without any problems.

## Conclusion

The excellent results of numerous studies indicate that Mg implants represent an important advancement in a long history of orthopedic implant development.

In addition to the excellent radiological results of surgically used Mg implants in previous publications, the intraoperative sites in a revision setting are displayed for the first time in this report. The homogeneous degradation with the good bone-implant interface, as well as the user-friendly operability, underline the special position of Mg in the field of resorbable metals.

## Funding

LORENZ BÖHLER Fond, Austria.

## Ethical approval

Approved by the ethics committee Graz, Austria (28-071 ex 15/16).

## Informed consent

Carried out after checking the inclusion and exclusion criteria.

## Conflict of interest

Patrick Holweg has received funding from LORENZ BÖHLER Fond, Austria.
